# Increased Unsaturation of Fatty Acids in Covalently Bound Ceramides Is Linked to Disordered Intercellular Lipid Structure and Skin Hypersensitivity

**DOI:** 10.1111/jocd.70489

**Published:** 2025-10-03

**Authors:** Akane Kawamoto, Taisei Joichi, Hiroaki Katsukura, Lili Zhai, Daisuke Watanabe, Naohito Yamamoto, Mai Haneoka, Shun Nakamura, Hiromitsu Nakazawa, Hiroyuki Yoshida

**Affiliations:** ^1^ Skin Care Products Research Kao Corporation Odawara Kanagawa Japan; ^2^ Analytical Science Research Kao Corporation Haga‐gun Tochigi Japan; ^3^ School of Science Kwansei Gakuin University Sanda Hyogo Japan

**Keywords:** barrier function, covalently bound ceramides, hypersensitivity, lipid organization, sensitive skin

**Abbreviations**
CE: cornified envelope
CER: ceramide
CER[NP]: non‐hydroxy fatty acid/phytosphingosine base ceramide
CER[NS]: non‐hydroxy fatty acid/sphingosine base ceramide
CER[OH]: ω‐hydroxy fatty acid/6‐hydroxy sphingosine base ceramide
CER[OS]: ω‐hydroxy fatty acid/sphingosine base ceramide
CLE: corneocyte lipid envelope
FA: fatty acid
ICL: intercellular lipid
LAST: lactic acid stinging test
MUFA: monounsaturated fatty acid
SC: stratum corneum
SFA: saturated fatty acid
SS: sensitive skin

To the Editor,

Sensitive skin (SS) type overreacts to external factors, causing unpleasant subjective symptoms like itching, burning, pain, and stinging [1]. SS‐like characteristics include decreased stratum corneum (SC) barrier function and abnormal neurosensory function [1]. However, the physiological and epidemiological characteristics of SS remain elusive.

The SC comprises several corneocyte layers surrounded by intercellular lipid (ICL) lamellae and a cornified envelope (CE). Non‐bound ceramides (CERs), which are the major components of ICL, and covalently bound CERs, which are crosslinked to CE proteins forming the corneocyte lipid envelope (CLE), are essential for maintaining SC barrier function [2]. Our recent study on healthy Japanese females (20–49 years) with SS and non‐SS reported that the SS subjects showed a decreased non‐bound CER [NP]/[NS] ratio compared with the non‐SS subjects, which was associated with the disordered lateral packing structure of ICLs and skin hypersensitivity in all the subjects [3]. However, all the correlation coefficients obtained were generally low, and therefore, there may be other factors affecting ICL packing and skin hypersensitivity in SS. Here, we additionally examined the amount, carbon chain length, and unsaturation of fatty acids (FAs) of covalently bound CERs using SC samples previously obtained [3], and their relationship with ICL structure and skin hypersensitivity.

The general characteristics of the study subjects with mild‐to‐moderate SS and non‐SS were summarized in our previous paper [3]. We first evaluated the levels of covalently bound CERs in the SC samples collected from the cheek of subjects with SS (*n* = 18) and non‐SS (*n* = 48) (Supporting Information and methods). As shown in Figure 1a,b, no significant differences were noted between SS and non‐SS in the levels of individual major species of covalently bound CERs, including CER[OH] with saturated FA (CER[OH]_Δ0) or monounsaturated FA (CER[OH]_Δ1) and CER[OS] with saturated FA (CER[OS]_Δ0) or monounsaturated FA (CER[OS]_Δ1), the level of total covalently bound CERs, and the carbon chain lengths of the four CERs. Thus, we further investigated the ratios of monounsaturated FAs (MUFAs) to saturated FAs (SFAs) (MUFA/SFA) in the CER[OH] and CER[OS]. We found that both MUFA/SFA ratios in the CER[OH] and CER[OS] were significantly higher in SS than those in non‐SS (Figure 1c).

**FIGURE 1:**
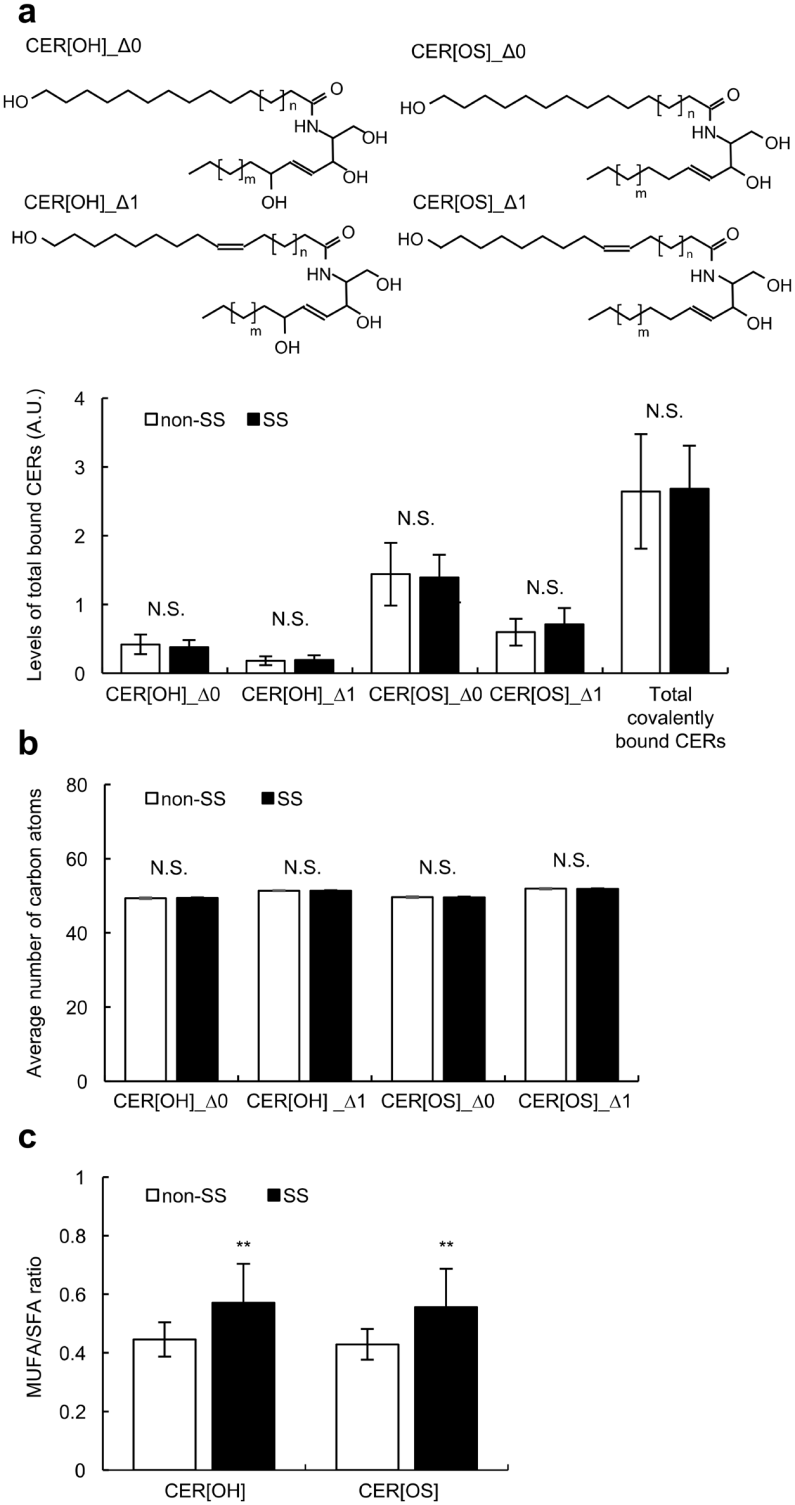
Amount, chain length, and unsaturation of fatty acids (FAs) of covalently bound ceramide (CER) species in subjects with sensitive skin (SS) and non‐SS. (a) Levels of CER species, including CER[OH]_Δ0, CER[OH]_Δ1, CER[OS]_Δ0, and CER[OS]_Δ1, and total covalently bound CERs in tape‐stripped stratum corneum (SC) from subjects with SS (*n* = 48) and non‐SS (*n* = 18). A.U., arbitrary unit. *Insets*, Representative structure of CER[OH]_Δ0, CER[OH]_Δ1, CER[OS]_Δ0, and CER[OS]_Δ1 (C44‐58:0 or C44‐58:1[Δ^9^]). (b) Average number of carbon atoms in CER[OH]_Δ0, CER[OH]_Δ1, CER[OS]_Δ0, and CER[OS]_Δ1 in tape‐stripped SC from subjects with SS (*n* = 48) and non‐SS (*n* = 18). (c) Ratios of monounsaturated FAs (MUFAs) to saturated FAs (SFAs) (MUFA/SFA) in CER[OH] and CER[OS] in tape‐stripped SC from subjects with SS (*n* = 48) and non‐SS (*n* = 18). Values are expressed as mean ± standard deviation (SD). Statistical significances were assessed using Student's *t*‐test. **p < 0.01. N.S., not significant.

We then investigated the relationship between the MUFA/SFA ratios in covalently bound CERs and lactic acid stinging test (LAST) score or the Pk2.7/Pk2.4 ratio, an indicator of the orthorhombic‐hexagonal lateral packing structure of ICLs. As shown in Figure 2a–d, the MUFA/SFA ratios in the CER[OH] and [OS] were positively and negatively correlated with the LAST scores and Pk2.7/Pk2.4 ratios, respectively. Because a decreased level of orthorhombic lateral packing contributes to higher SC permeability [4], increased MUFA/SFA ratios in covalently bound CERs may be involved in the disordered lateral packing of ICLs, which may be related to hypersensitivity to lactic acid. Furthermore, both MUFA/SFA ratios in the CER[OH] and CER [OS] were negatively correlated with the corneocyte size (Figure 2e–g). As corneocyte size is reduced in inflamed skin [5], we hypothesized that low‐level inflammation may occur in SS and be one possible reason for the increased unsaturation of FAs. Previously, stearoyl‐coenzyme A desaturase‐1, an enzyme involved in forming unsaturated FAs from saturated FAs, was reported to increase in inflamed atopic dermatitis skin [6]. Therefore, to confirm the hypothesis, further studies would be worthwhile to investigate the involvement of this enzyme in the increased unsaturation of FAs observed in SS.

**FIGURE 2:**
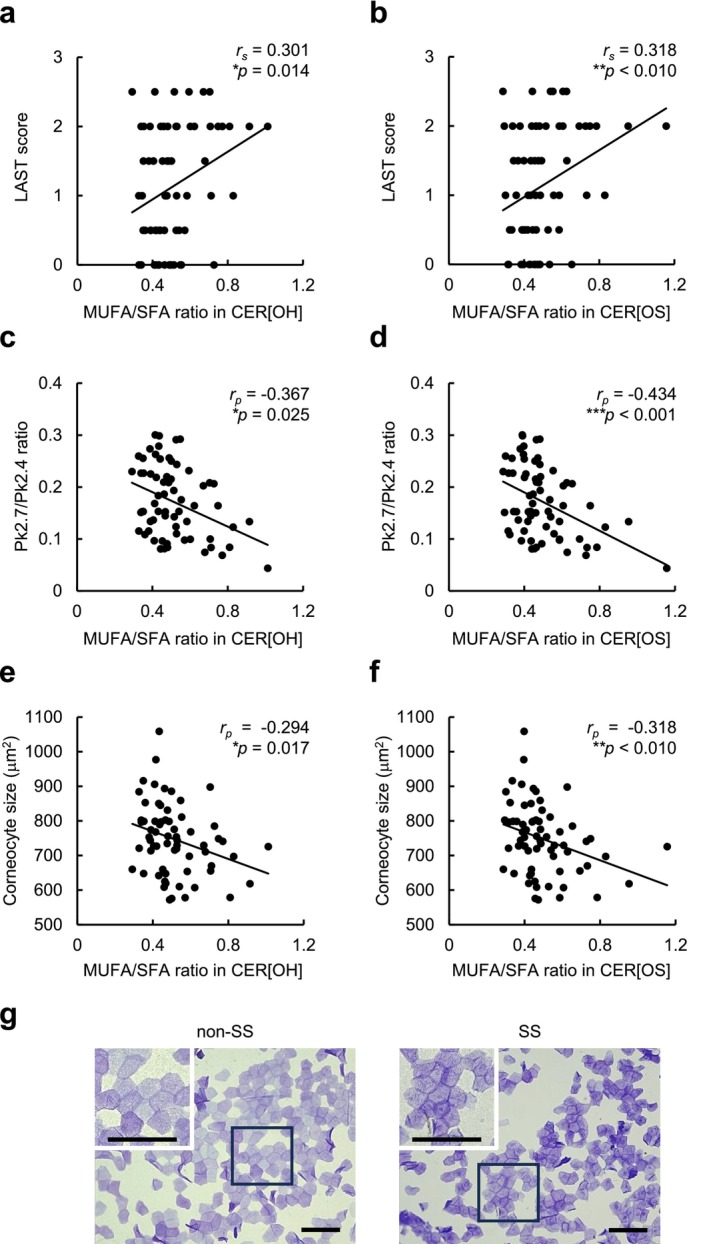
Correlations of MUFA/SFA ratios in CER[OH] and CER[OS] with lactic acid stinging test (LAST) score, Pk2.7/Pk2.4 ratio, and corneocyte size. (a and b) Correlations between MUFA/SFA ratios in CER[OH] (a) or CER[OS] (b) and LAST score in all subjects (*n* = 66). (c and d) Correlations between MUFA/SFA ratios in CER[OH] (c) or CER[OS] (d) and Pk2.7/Pk2.4 ratio in all subjects (*n* = 66). (e and f) Correlations between MUFA/SFA ratios in CER[OH] (e) or CER[OS] (f) and corneocyte size in all subjects (*n* = 66). (g) Representative images of tape‐stripped corneocytes from subjects with SS and non‐SS stained with gentian violet and brilliant green. *Insets*, high‐power view of boxed areas. Scale bars = 100 μm. Correlations were examined using Spearman's correlation analysis for (a) and (b) or Pearson's correlation analysis for (c–f). *r*_*s*_, Spearman's correlation coefficient; *r*_*p*_, Spearman's correlation coefficient. *p < 0.05; **p < 0.01; ***p < 0.001.

A limitation of this study includes a small sample size, and further studies are needed to generalize our conclusions. However, taken together with our previous findings [3], abnormalities not only in non‐bound CERs but also in covalently bound CERs are linked to disordered intercellular lipid structures and skin hypersensitivity.

## Author Contributions

A.K., T.J., D.W., N.Y., and H.Y. designed research; A.K., T.J., H.K., L.Z., D.W., and M.H. performed research; A.K., T.J., H.K., L.Z., D.W., N.Y., M.H., S.N., H.N., and H.Y. analyzed data; A.K., M.H., and H.Y. wrote the manuscript; A.K. and H.Y. reviewed and revised the manuscript. The authors approved the final manuscript.

## Ethics Statement

This study was approved by the Ethics Committee of Kao Corporation (study number: D164‐210115) and conducted following the Declaration of Helsinki. Informed consent was obtained from all the subjects.

## Conflicts of Interest

The authors declare no conflicts of interest.

## Supporting information


**Data S1:** Supplementary Information.

## Data Availability

The data that support the findings of this study are available on request from the corresponding author. The data are not publicly available due to privacy or ethical restrictions.
