# Supplementary material for: Increased Unsaturation of Fatty Acids in Covalently Bound Ceramides Is Linked to Disordered Intercellular Lipid Structure and Skin Hypersensitivity
Source: J Cosmet Dermatol. 2025 Oct 3;24(10):e70489. doi: 10.1111/jocd.70489 (PMC12495121; doi:10.1111/jocd.70489)
Supplement: Supplementary file 1 — Data S1: Supplementary Information. [file JOCD-24-e70489-s001.docx]

**Supporting materials and methods**

***Screening of study subjects***

This study was approved by the Ethics Committee of Kao Corporation (study number: D164-210115) and conducted following the Declaration of Helsinki. Informed consent was obtained from all the subjects. One hundred seventy healthy Japanese females (20–49 years) participated in this study. According to their responses to a self-assessment questionnaire about facial skin sensitivity, 37 individuals were enrolled in the self-perceived non-sensitive skin (SS) group, and the remaining 133 were enrolled in the self-perceived SS group. Based on 1% lactic acid stinging test (LAST) scores obtained as described below, 18 individuals (LAST score < 1) in the self-perceived non-SS group were classified as subjects without SS (mean age ± standard deviation [SD], 35.11 ± 10.49 years), and 48 individuals (LAST score ≥ 1) in the self-perceived SS group were classified as subjects with SS (mean age ± SD, 36.52 ± 8.63 years). Subjects with skin diseases, including congenital ichthyosis, atopic dermatitis, pregnancy, lactation, or severe allergies were excluded. Prior to the measurement of skin parameters, all subjects washed their faces and acclimated for 15 min in a room with constant humidity (49.5 ± 4.5%) and temperature (21.5 ± 1.0°C).

***LAST***

LAST was conducted with a piece of non-woven cloth (20 mm × 50 mm square) soaked with 400 μL of aqueous solution containing 1% lactic acid and distilled water heated to 32°C. Distilled water was applied by placing a cloth on the left cheek. The subjects reported the intensity of the sensation as itching, burning, pain, or crawling using a 4-point scale (none = 0, mild = 1, moderate = 2, severe = 3) at 0.5, 2.5, and 5 min after application. Half-intermediate scores were used for evaluation. After 5 min, a 1% lactic acid solution was applied to the same area, and the subjects reported the intensity of the sensation. The LAST scores were calculated using a previously described formula.^1^

***Analysis of covalently bound ceramides***

Stratum corneum (SC) sheets were sequentially collected from the right cheek by tape stripping. After applying the tape to the skin, the tape was gently stroked for five times using the pad of the index finger, then slowly peeled off while keeping it aligned with the skin. The first sheet was discarded and the second to fourth sheets were used for the analysis. After extracting non-bound ceramides (CERs), SC flakes were dispersed from each tape sample and treated with alkaline to release covalently bound CERs. CERs were analyzed by liquid chromatography/mass spectrometry (LC/MS) using an Agilent LC/MSD (6125 B) system equipped with an electrospray ionization (ESI) source, ChemStation software, and an Agilent 1260 Infinity II Series LC system (Agilent Technologies, Santa Clara, CA, USA), as previously described.^2^ Covalently bound CERs were relatively quantified by the area ratio to the internal standard (N-heptadecanoyl-D-erythro-sphingosine [Avanti Polar Lipids, Alabaster, AL, USA]). The quantitative values of each CER subclass were normalized by the protein amount of each tape-stripped SC sample measured by bicinchoninic acid (BCA) protein assay.^2^ The relative amounts of covalently bound CERs (the area ratio to the internal standard normalized per protein amount) were used for statistical analysis.

***Analysis of the lateral packing structure of intercellular lipids***

SC samples were collected from the right cheek using the grid-stripping method, and the lateral packing structure of intercellular lipids (ICLs) was analyzed using low-flux electron diffraction.^3-6^ Corneocytes sticking to the grid were examined using a conventional transmission electron microscope (JEM1400, JEOL) operated at an acceleration voltage of 100 kV. Electron diffraction images of selected areas of 55 μm^2^ were obtained from at least 24 corneocytes per subject. According to a previous study, the electron dose for an image was approximately 1.4 e∙nm^-2^, which is small enough not to damage the structural organization of ICLs.^6^ For quantitative analysis, electron diffraction intensity profiles were calculated as a function of the scattering vector (*s* = 2sinθ/*λ*) by integrating the intensity along the azimuthal direction. The peak intensity ratio (Pk2.7/Pk2.4) of the diffraction peaks at s ≈ 2.4 nm^-1^ and s ≈ 2.7 nm^-1^ derived from hexagonal and orthorhombic packing structures was evaluated to clarify the difference in packing structure of ICLs between subjects with SS and non-SS.^5^

***Measurement of corneocyte size***

Tape-stripping was performed on the right cheek using a CAF sheet (Toppan, Tokyo, Japan). The stripped SC sheets were immersed for 10 min in an aqueous solution containing 1% gentian violet (FUJIFILM Wako Pure Chemical, Osaka, Japan) and 0.5% brilliant green (FUJIFILM Wako Pure Chemical).^7^ After washing with water, the sheets were dried and sealed on a glass slide with Malinol 750cps (MUTO PURE CHEMICALS, Tokyo, Japan). Corneocyte size was analyzed using images captured by microscopy with corneocytometry 2.0 (CIEL, Osaka, Japan).^7^

***Statistics***

Statistical significance was assessed using Student's *t*-test, and correlations were examined using Spearman’s or Pearson’s correlation analysis with IBM SPSS Statistics 28.0 (IBM, Armonk, NY, USA) or Excel (Microsoft 365) (Microsoft, Redmond, WA, USA). Statistical significance was set at *p <* 0.05.

**References**

1. Yatagai T, Shimauchi T, Yamaguchi H et al. Sensitive skin is highly frequent in extrinsic atopic dermatitis and correlates with disease severity markers but not necessarily with skin barrier impairment. *J Dermatol Sci.* 2018;89(1):33-39. doi:[10.1016/j.jdermsci.2017.10.011](https://doi.org/10.1016/j.jdermsci.2017.10.011).

2. Kawamoto A, Yoshida H, Haneoka M et al. Chain length of covalently bound ceramides correlates with skin barrier function in healthy subjects. *J Dermatol Sci.* 2023;110(1):35-38. doi:[10.1016/j.jdermsci.2023.02.007](https://doi.org/10.1016/j.jdermsci.2023.02.007).

3. Pilgram GS, Engelsma-van Pelt AM, Oostergetel GT et al. Study on the lipid organization of stratum corneum lipid models by (cryo-) electron diffraction. *J Lipid Res.* 1998;39(8):1669-1676. doi:[10.1016/S0022-2275(20)32197-0](https://doi.org/10.1016/S0022-2275(20)32197-0).

4. Pilgram GS, Vissers DC, van der Meulen H et al. Aberrant lipid organization in stratum corneum of patients with atopic dermatitis and lamellar ichthyosis. *J Invest Dermatol.* 2001;117(3):710-717. doi:[10.1046/j.0022-202x.2001.01455.x](https://doi.org/10.1046/j.0022-202x.2001.01455.x).

5. Nakazawa H, Imai T, Hatta I et al. Low-flux electron diffraction study on body site dependence of stratum corneum structures in human skin. *Biochim Biophys Acta Biomembr.* 2022;1864(9):183933. doi:[10.1016/j.bbamem.2022.183933](https://doi.org/10.1016/j.bbamem.2022.183933).

6. Nakazawa H, Imai T, Hatta I, et al. Low-flux electron diffraction study for the intercellular lipid organization on a human corneocyte. *Biochim Biophys Acta.* 2013;1828(6):1424-1431. doi:[10.1016/j.bbamem.2013.02.001](https://doi.org/10.1016/j.bbamem.2013.02.001).

7. Doi M, Sagawa Y, Momose S, et al. Topical treatment with sacran, a sulfated polysaccharide from Aphanothece sacrum, improves corneocyte-derived parameters. *J Dermatol.* 2017;44(12):1360-1367. doi:[10.1111/1346-8138.13970](https://doi.org/10.1111/1346-8138.13970).
